# Modulating social learning-induced evaluation updating during human sleep

**DOI:** 10.1038/s41539-024-00255-5

**Published:** 2024-07-07

**Authors:** Danni Chen, Tao Xia, Ziqing Yao, Lingqi Zhang, Xiaoqing Hu

**Affiliations:** 1 Department of Psychology, The State Key Laboratory of Brain and Cognitive Sciences, The University of Hong Kong, Hong Kong SAR, China; 2 HKU-Shenzhen Institute of Research and Innovation, Shenzhen, China

**Keywords:** Human behaviour, Learning and memory

## Abstract

People often change their evaluations upon learning about their peers’ evaluations, i.e., social learning. Given sleep’s vital role in consolidating daytime experiences, sleep may facilitate social learning, thereby further changing people’s evaluations. Combining a social learning task and the sleep-based targeted memory reactivation technique, we asked whether social learning-induced evaluation updating can be modulated during sleep. After participants had indicated their initial evaluation of snacks, they learned about their peers’ evaluations while hearing the snacks’ spoken names. During the post-learning non-rapid-eye-movement sleep, we re-played half of the snack names (i.e., cued snack) to reactivate the associated peers’ evaluations. Upon waking up, we found that the social learning-induced evaluation updating further enlarged for both cued and uncued snacks. Examining sleep electroencephalogram (EEG) activity revealed that cue-elicited delta-theta EEG power and the overnight N2 sleep spindle density predicted post-sleep evaluation updating for cued but not for uncued snacks. These findings underscore the role of sleep-mediated memory reactivation and the associated neural activity in supporting social learning-induced evaluation updating.

## Introduction

Evaluations and choices are often guided by retrieval of first-hand experiences: when choosing a restaurant, we often think about our last visit, the dining experiences, and the accompanying emotional feelings^1–3^. However, in addition to using first-hand experiences to guide our choices^4–6^, we also acquire or change evaluations via observing our peers’ evaluations and choices, known as social learning^7–9^. Social learning is prevalent in society, influencing everyday choices, such as purchasing snacks or books, and even sacred moral values^10–13^. Specifically, social learning can be induced in lab settings: following observing peers’ evaluations, participants often change their initial evaluations^11,13–15^. These social learning-induced evaluation updating can even last for days after the learning^15,16^. The observed long-term effect raises an intriguing yet untested question: how does memory consolidation during post-learning sleep influence the social learning effect?

Mounting evidence suggests that sleep consolidates recently acquired memories via covert memory reactivation processes^17–19^. Employing a method known as Targeted Memory Reactivation (TMR), researchers can initiate and guide covert memory reactivation during sleep to promote memory consolidation^20,21^. This TMR procedure typically consists of three phases: (1) pre-sleep learning, participants would learn materials accompanying sensory cues (e.g., auditory tones, spoken words, odor); (2) TMR during sleep, during which the experimenter will re-present the same sensory cues (i.e., memory reminders) to sleeping participants to reactivate the associated memories; and (3) post-sleep tests, upon awakening, participants would complete tests to assess the impact of TMR. Accumulating evidence has demonstrated that TMR benefits various types of memories (for a meta-analysis, see Hu et al.^22^), including speech-word pair associative learning^23^, skills learning^24,25^, spatial memories^26,27^, and emotional memories^28,29^. Here, we aimed to explore the potential impact of TMR on people’s evaluations acquired through prior social learning.

To date, only a few studies have explored the potential impact of sleep and/or TMR on evaluation. For example, sleep (vs. wakefulness) promoted adaptive evaluative choices, by strengthening evaluative learning memories^30^. Employing TMR, research shows that re-playing snacks’ spoken names during non-rapid eye movement (NREM) sleep could augment subjective preferences for these snacks^31^. Moreover, replaying the sound cues paired with the prior counter-bias training during NREM sleep further reduced implicit social biases^32^ (but see ref. ^33^). These findings suggest that sleep and/or TMR, via sleep-mediated reactivation of pre-sleep evaluative memories, could modulate post-sleep evaluations and choices

Analyzing cue-elicited electroencephalogram (EEG) activity during sleep can provide insights into the underlying neural mechanisms of TMR. Notably, cue-elicited delta (1–4 Hz) and theta (4–8 Hz) activities have been shown to predict TMR benefits on memory performance^34–38^. More specifically, research also revealed the role of cue-elicited delta and theta power in predicting TMR benefits in evaluation updating^31,39^. Furthermore, the sleep spindles are pivotal in memory re-processing during sleep, with cueing-related spindle activities predicting TMR benefits^28,40–45^. We thus focused on the delta/theta power and the sleep spindles underlying the reactivation of daytime social learning experiences.

**Fig. 1:**
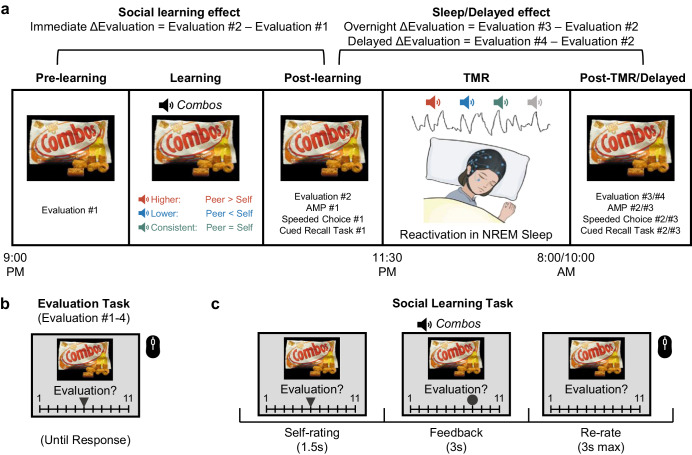
A flowchart of the experiment procedure. **a** The experiment included pre-learning baseline tests, a social learning task in which participants learned their peers’ evaluations, post-learning immediate tests, TMR during NREM sleep, post-TMR tests, and 3-day delayed tests. We determined the immediate ΔEvaluation as the difference between pre-and post-learning, overnight ΔEvaluation as the difference between post-learning and post-TMR, and delayed ΔEvaluation as the difference between post-learning and delayed phases. **b** An exemplar trial in the Evaluation tasks: Participants evaluated each of the 48 snacks using a mouse clicking on a 1-11 scale, ranging from not preferred at all (1) to most preferred (11). **c** During the Social Learning task, participants learned the evaluation from their peers (a circle indicating their peers’ evaluation) while hearing the spoken names of the snacks upon the onset of the peers’ evaluations. Half of these auditory cues were then re-played during the following NREM sleep to reactivate the social learning memories (i.e., peers’ evaluation toward the snack). This resulted in six experimental conditions (Higher_Cued vs. Uncued; Lower_Cued vs. Uncued; Consistent_Cued vs. Uncued). The snack picture is from Hare et al.^74^.

Here, we employed the TMR to investigate how reactivating prior social learning experiences during NREM sleep would influence social learning-induced evaluation updating (Fig. 1). Following the initial evaluation for snacks, participants learned their peers’ evaluations as feedback while listening to the snacks’ spoken names. These spoken names would serve as memory reminders about peers’ evaluations of the snacks. During the subsequent NREM sleep, we replayed half of the snacks’ spoken names to reactivate their associated peers’ evaluations (i.e., TMR). Upon waking up, participants showed enlarged social learning-induced evaluation updating for both cued and uncued snacks. Accompanying behavioral changes, cue-elicited delta-theta EEG power, and the overnight N2 spindle density, were associated with the evaluation updating for cued but not for uncued snacks. These results suggested that sleep-mediated memory reactivation processes fortify social learning-induced evaluation updating.

## Results

### Effects of social learning and TMR on evaluation updating

**Fig. 2:**
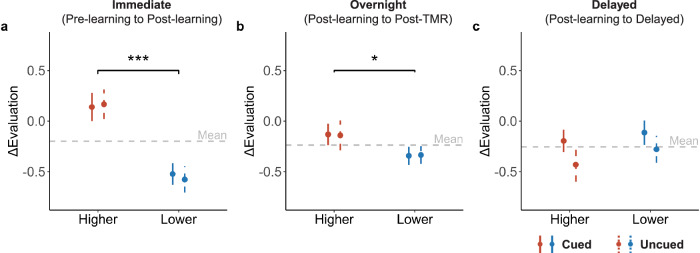
Impact of feedback and TMR on evaluation updating across phases. Effects of feedback (i.e., peers’ ratings either higher or lower than pre-learning baseline ratings) and TMR (cued vs. uncued) on ΔEvaluation from (**a**) pre-learning to post-learning, (**b**) post-learning to post-TMR, and (**c**) post-learning to delayed phases. The error bars indicate the standard error of the mean (S.E.M.). The horizontal gray dashed line represents the mean of ΔEvaluation at the corresponding phase. ***: *p* < 0.001. *: *p* < 0.05.

We began by examining whether social learning modulated evaluations of the snacks. In a TMR (cued vs. uncued) by feedback (higher vs. lower) repeated measure ANOVA, we found the expected social learning effect: feedback significantly modulated immediate ΔEvaluation (i.e., changes of evaluation from pre- to post-learning; *F* (1, 33) = 23.42, *p* < 0.001, ${\eta }_{G}^{2}$ = 0.18; Fig. 2a). Specifically, when peers’ evaluations were higher than participants’ initial evaluations, participants’ evaluations increased accordingly. In contrast, the TMR effect was not significant (*F* (1, 33) = 0.02, *p* = 0.877, ${\eta }_{G}^{2}$ < 0.01) nor was the TMR by feedback interaction (*F* (1, 33) = 0.34, *p* = 0.564, ${\eta }_{G}^{2}$ < 0.01), indicating that cued and uncued snacks showed comparable social learning effects before sleep and TMR manipulation.

We next examined the impact of sleep TMR on the overnight ΔEvaluation from the post-learning to post-TMR phase. We again found a significant main effect of feedback, such that the ΔEvaluation was significantly increased for the higher than for the lower feedback condition (*F* (1, 33) = 4.72, *p* = 0.037, ${\eta }_{G}^{2}$ = 0.03; Fig. 2b). This significant feedback effect on overnight ΔEvaluation indicated that the difference between higher vs. lower feedback directions further enlarged from post-learning to post-TMR phases. Contrary to our hypotheses, neither the TMR (cued vs. uncued) nor the TMR by feedback interaction was significant (*F* (1, 33) < 0.01, *p* = 0.994, ${\eta }_{G}^{2}$ < 0.01; *F* (1, 33) = 0.01, *p* = 0.911, ${\eta }_{G}^{2}$ < 0.01, respectively).

We further examined the 3-day delay effect of sleep TMR on the delayed ΔEvaluation from post-learning to the 3-day delayed phase. We found a non-significant trend of the TMR effect: cued snacks showed numerically higher ΔEvaluation than uncued snacks (*F* (1, 33) = 3.69, *p* = 0.063, ${\eta }_{G}^{2}$ = 0.02; Fig. 2c). Neither feedback (*F* (1, 33) = 1.23, *p* = 0.275, ${\eta }_{G}^{2}$ = 0.01) nor interaction effects (*F* (1, 33) = 0.18, *p* = 0.677, ${\eta }_{G}^{2}$ < 0.01) were significant. We postulated that the cueing might increase familiarity, thus enhancing preferences^31^. Indeed, in a TMR by feedback repeated measure ANOVA on the familiarity rating, we found that cueing significantly enhanced familiarity ratings of snacks in the 3-day delayed session (*F* (1, 33) = 8.28, *p* = 0.007, ${\eta }_{G}^{2}$ = 0.03), but not in the post-learning nor post-TMR tests (*p*s > 0.116). Thus, the numerically higher evaluations of cued snacks could be attributed to their higher familiarity at the delayed phase.

### Effects of social learning and TMR on memory errors

Here, we examined whether TMR changed memory errors, i.e., the absolute numerical differences between participants’ recalled peers’ ratings and the presented peers’ ratings. In the TMR by feedback repeated measure ANOVA, we did not find a significant main or interaction effect in the post-learning phase (*p*s > 0.487). In the post-TMR phase, we observed a non-significant trend of increased memory error for the higher than the lower feedback conditions (*F* (1, 33) = 4.01, *p* = 0.054, ${\eta }_{G}^{2}$ = 0.02). However, no significant main effect of TMR (*F* (1, 33) = 0.96, *p* = 0.333, ${\eta }_{G}^{2}$ < 0.01), and the interaction effect was observed (*F* (1, 33) = 0.02, *p* = 0.879, ${\eta }_{G}^{2}$ < 0.01). In the delayed phase, no significant main effects nor interaction effects were found (*p*s > 0.230).

### Relationship between subsequent memory accuracies and evaluation updating

Although TMR did not influence memory errors when recalling peers’ evaluative ratings, we examined whether evaluation updating was associated with memory accuracies, i.e., whether participants’ recall of the peers’ ratings aligned with the feedback directions. To examine this question, we conducted feedback by TMR by subsequent memory (correctly vs. incorrectly remembered) three-way item-level BLMM for ΔEvaluation.

**Fig. 3:**
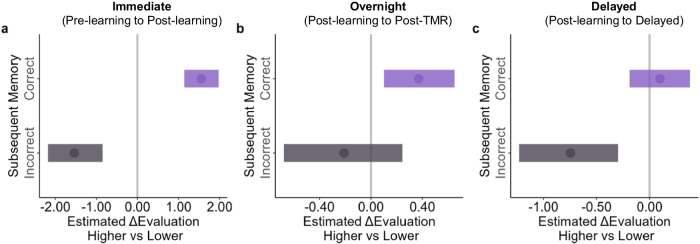
Impact of subsequent memory, feedback and TMR on evaluation updating across phases. Effects of subsequent memory, TMR, and feedback on ΔEvaluation from (**a**) pre-learning to post-learning, (**b**) post-learning to post-TMR, and (**c**) post-learning to delayed phases. The horizontal lines indicated the 95% highest density interval (HDI), and the vertical gray lines correspond to 0. The dot indicates the median. If the 95% HDI does not encompass 0, the result is significant.

For the immediate ΔEvaluation from pre-learning to post-learning, we found a significant interaction between subsequent memory and feedback (median = 2.94, 95% HDI [1.93, 3.85], Fig. 3a). Post-hoc analysis revealed that when participants correctly remembered the feedback direction, the immediate ΔEvaluation in the higher feedback condition was significantly higher than that in the lower feedback condition (higher vs. lower, median_*diff*_ = 1.55, 95% HDI [1.14, 1.98]). Conversely, when participants incorrectly remembered the feedback direction, the immediate ΔEvaluation in the higher feedback condition was significantly lower than in the lower feedback condition (median_*diff*_ = -1.54, 95% HDI [-2.18, -0.85]).

For the overnight ΔEvaluation from post-learning to post-TMR, we similarly found a significant subsequent memory by feedback interaction (median= 0.74, 95% HDI [0.03, 1.46], Fig. 3b). Post-hoc analyses revealed that when participants correctly remembered the feedback direction, the overnight ΔEvaluation in the higher feedback condition was significantly higher than that in the lower condition (median_*diff*_ = 0.37, 95% HDI [0.10, 0.65]). In contrast, when participants incorrectly remembered the feedback direction, the overnight ΔEvaluation did not differ between the higher and the lower condition (median_*diff*_ = −0.21, 95% HDI [−0.68, 0.25]).

For the delayed ΔEvaluation from post-learning to the delayed phase, the same BLMM again revealed a significant interaction effect (median_*diff*_ = 0.71, 95% HDI [0.01, 1.40], Fig. 3c). Post-hoc analyses revealed that when participants correctly remembered the feedback direction, the ΔEvaluation between the higher and the lower condition did not differ (median_*diff*_ = 0.10, 95% HDI [-0.19, 0.38]). In contrast, when participants incorrectly remembered the feedback direction, the ΔEvaluation of the higher condition was significantly lower than that in the lower condition (median_*diff*_ = -0.75, 95% HDI [-1.23, -0.30]). These results suggested that the evaluation updating was related to the memory of the feedback directions across all three phases.

### Effects of social learning and TMR on implicit evaluation and speeded choice

Observing the social learning effects on subjective evaluation updating, we further examined whether social learning and TMR could impact implicit evaluation (ΔImplicit evaluation based on AMP performance) and speeded choices (Δ%Choose based on the speeded choice task) by conducting TMR by feedback repeated measure ANOVAs.

In the speeded choice task, we observed a significant main effect of feedback in overnight Δ%Choose from post-learning to post-TMR phases: participants chose more snacks in the higher than the lower feedback conditions (*F* (1, 32) = 4.83, *p* = 0.035, ${\eta }_{G}^{2}$ = 0.03). No significant effect of TMR nor their interaction was found (*p*s >.316; Supplementary Fig. 1a). Similarly, no significant effect of feedback, TMR, nor their interaction in delayed Δ%Choose was observed (*p*s > 0.283; Supplementary Fig. 1b).

In the AMP, we did not observe a significant effect of feedback, TMR, nor their interaction in the ΔImplicit evaluation from post-learning to post-TMR (*p*s > 0.312; Supplementary Fig. 1c) and to delayed phases (*p*s > 0.398; Supplementary Fig. 1d).

### Cue-elicited delta-theta power predicted evaluation updating of cued snacks

Even though we did not observe the TMR effect on ΔEvaluation during the post-TMR phases, we proceeded to investigate how sleep EEG changes may drive the overall enhanced social learning effect for both cued and uncued snacks.

**Fig. 4:**
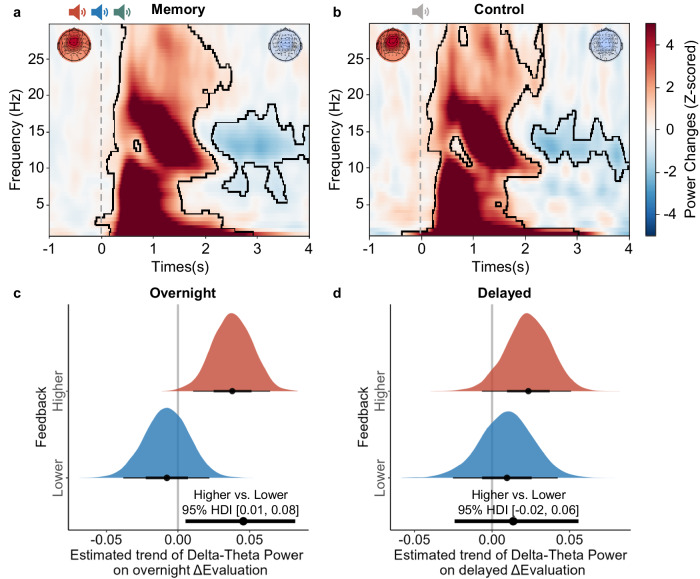
Cue-elicited EEG Power and ΔEvaluation. **a** Memory cue (higher, lower, and consistent) and (**b**) control cue-elicited power spectral averaged across nine fronto-central channels (F1/2, Fz, FC1/2, FCz, C1/2, Cz). The topography on the left-top and right-top corners indicated the power at all 61 channels at the early and late clusters, respectively. The contour highlighted significant clusters. The effect of memory cue-elicited delta-theta power (1–8 Hz) on ΔEvaluation of cued snacks from (**c**) post-learning to post-TMR and (**d**) post-learning to delayed phases. The black line below the red and blue density plots indicated the 95% highest density interval (HDI) for higher and lower feedback conditions, respectively. The bottom black line indicates the difference between higher vs. lower feedback conditions. The dot indicates the median point. If the 95% HDI does not encompass 0, the result is considered significant.

We first examined whether presenting cues during sleep would elicit significant EEG power changes relative to the pre-cue baseline (i.e., −1000 to −200 ms prior to the cue onset). For this purpose, we performed time-frequency analyses on artifact-free EEG epochs and conducted a cluster-based two-tailed one-sample permutation test across time and frequency bands, based on the EEG power averaged across feedback conditions and across the pre-defined fronto-central electrodes (F1/2, Fz, FC1/2, FCz, C1/2, Cz; see Methods for details; Supplementary Fig. 2). We found that the cues significantly enhanced the 1-30 Hz power during an early cluster (-96 to 2928 ms relative to the cue onset, *p*_*cluster*_ = 0.001, corrected for multiple comparisons by cluster-based permutation test) but reduced the 5.5–18.5 Hz power in a later cluster (2132–4000 ms, *p*_*cluster*_ = 0.025, Fig. 4a). However, we did not find significant EEG power differences between the higher and lower feedback conditions (*p*_*cluster*_s > 0.085, Supplementary Fig. 3a–c). Similarly, the control cues enhanced the 1-30 Hz EEG power in the early cluster (−360 to 3028 ms relative to the cue onset, *p*_*cluster*_ = 0.001) but reduced the 8.5–17.5 Hz power in the later cluster (2136–4000 ms, *p*_*cluster*_ = 0.047, Fig. 4b). However, further analysis did not reveal significant EEG differences between memory and control cues (*p*_*cluster*_s > 0.217, Supplementary Fig. 3d). These results suggested that both memory and control cues were processed during sleep.

Employing the item-level BLMM, we next examined whether memory cue-elicited EEG power could predict the ΔEvaluation of cued snacks. We extracted cue-elicited delta-theta power (1–8 Hz) and sigma power (12–16 Hz) within the 0–2 s of the early identified cluster at the item level. We selected the 0–2 s time window because it captured the early cluster yet did not overlap with the late cluster. The delta-theta EEG power by feedback BLMM on overnight ΔEvaluation (from post-learning to post-TMR phase) showed a significant interaction (higher vs. lower, median_*diff*_ = 0.05, 95% HDI [0.01, 0.08], Fig. 4c): cue-elicited delta-theta power predicted the post-TMR overnight evaluation updating for cued snacks as a function of feedback. Post-hoc analyses showed that cue-elicited delta-theta power significantly predicted ΔEvaluation (median = 0.04, 95% HDI [0.01, 0.06]) in the higher, but not in the lower feedback condition (median = −0.01, 95% HDI [−0.04, 0.02]). Furthermore, we conducted a subject-level BLMM analysis using cue-elicited delta-theta power, feedback (higher vs. lower), and their interaction as fixed variables to predict overnight ΔEvaluation of uncued snacks. However, this analysis did not yield significant predictions (higher vs. lower, median_*diff*_ = −0.00, 95% HDI [−0.04, 0.03]). Together, these results indicated that for cued snacks, higher cue-elicited delta-theta power predicted larger increase in evaluations in the higher feedback condition.

We next examined whether control cue-elicited delta-theta power would predict overnight ΔEvaluation. Using subject-level BLMM analyses including TMR (cued vs. uncued), feedback (higher vs. lower), and control cue-elicited delta-theta power as fixed factors, we did not find significant predictions for either cued or uncued snacks (−0.02 < median_*diff*_s < 0.02, all 95% HDIs overlap with 0).

Regarding delayed ΔEvaluation, neither memory cue- nor control cue-elicited delta-theta predicted ΔEvaluation (−0.02 < median_*diff*_s < 0.01, all 95% HDIs overlap with 0, see Fig. 4d). Additionally, cue-elicited sigma power did not predict both overnight and delayed ΔEvaluation (−0.00 < median_*diff*_s < 0.04, all 95% HDIs overlap with 0, Supplementary Fig. 4a, b).

### Overnight N2 sleep spindle density predicted evaluation updating for cued snacks

Given the sleep spindle’s crucial role in sleep-mediated memory consolidation^41^, we further examined the relationship between cued-elicited and overnight spindle activities and the evaluation updating.

First, we examined whether the cue elicited spindles relative to the [−1000 to 0 ms] pre-cue baseline. A cluster-based two-tailed one-sample permutation test on spindle probabilities revealed that both memory (-24 ~ 1920 ms; *p*_cluster_ = 0.001) and control cues (−148 ~ 1788 ms; *p*_cluster_ = 0.001) elicited significantly higher spindle probabilities than the pre-stimulus baseline (Supplementary Fig. 4c). However, no significant differences were found between memory vs. control cue-elicited spindle probability or among the different feedback conditions within memory cues (*p*_cluster_ > 0.707, Supplementary Fig. 4d).

Next, we extracted the item-level cue-elicited spindle probabilities by averaging the spindle probabilities within the significant [0, 1920 ms] time window across cue repetitions. The cue-elicited spindle probabilities were baseline-corrected by subtracting the mean of baseline spindle probabilities from the extracted probabilities. We then conducted an item-level BLMM to investigate whether cue-elicited spindle probabilities predicted ΔEvaluation, with feedback and spindle probabilities as fixed factors and the number of cue repetitions as a covariate. The results showed that cue-elicited spindle probability did not predict overnight (higher vs. lower, median_*diff*_ = −0.40, 95% HDI [−3.71, 2.75]) nor delayed (median_*diff*_ = −0.75, 95% HDI [−4.31, 2.62]) ΔEvaluation for the cued snacks (Supplementary Fig. 4e, f).

As we observed overnight evaluation updating for both cued and uncued snacks, we further explored the associations between overnight N2 spindle density and overnight ΔEvaluation (post-TMR minus post-learning) using a subject-level BLMM. In this model, TMR (cued vs. uncued), feedback (higher vs. lower), and overnight N2 spindle density were treated as fixed factors, allowing us to directly compare the effects of overnight spindle activities on cued and uncued snacks.

**Fig. 5:**
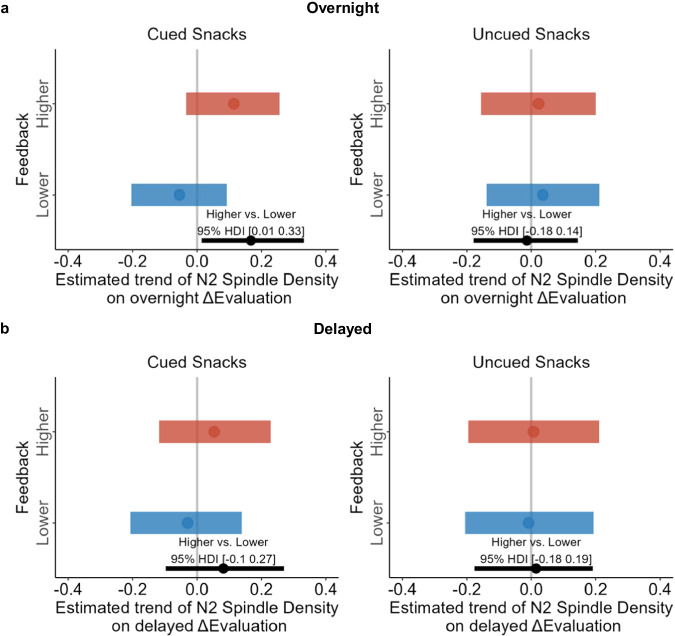
Relationship between overnight N2 spindle density and evaluation updating across phases. The relationship between overnight N2 Spindle Density and (**a**) overnight and (**b**) delayed ΔEvaluation. The left figure shows the effect on the cued snacks, while the right figure represents the effect on the uncued snacks. The vertical gray lines correspond to 0. The horizontal red and blue lines indicated the 95% highest density interval (HDI) for higher and lower feedback conditions, respectively. The bottom black line indicates the difference in higher vs. lower feedback conditions. The dot indicates the median. If the 95% HDI does not encompass 0, the result is considered significant.

The results showed that for cued snacks, overnight N2 spindle density differentially predicted overnight ΔEvaluation for higher and lower feedback conditions (higher vs. lower, median_*diff*_ = 0.17, 95% HDI [0.01, 0.33]; Fig. 5a). Specifically, increased overnight spindle density was associated with enhanced overnight evaluation for the higher feedback condition yet with reduced evaluation in the lower feedback condition. In contrast, this effect was not significant for uncued snacks (higher vs. lower, median _*diff*_ = −0.01, 95% HDI [−0.18, 0.14]; Fig. 5a). However, the same BLMM did not predict delayed ΔEvaluation for either cued (higher vs. lower, median_*diff*_ = 0.08, 95% HDI [−0.10, 0.27]; Fig. 5b) or uncued snacks (higher vs. lower, median_*diff*_ = 0.01, 95% HDI [−0.18, 0.19]; Fig. 5b).

To understand the function of N2 spindles in the evaluation updating, we further explored whether spindle density during the early (first three hours of sleep, overlapping with the TMR cueing) and late N2 (after three hours until the next morning wakefulness, following TMR cueing) would predict overnight ΔEvaluation. In this model, we included time (early vs. late), TMR (cued vs. uncued), feedback (higher vs. lower), and N2 spindle density as fixed factors. The results showed that both early and late N2 spindle density predicted overnight ΔEvaluation of cued snacks differently for higher and lower feedback conditions (higher vs. lower; early: median_*diff*_ = 0.17, 95% HDI [0.01, 0.33]; late: median_*diff*_ = 0.16, 95% HDI [0.03, 0.28]; Supplementary Fig. 5a, c). However, neither early nor late spindle density predicted overnight ΔEvaluation for uncued snacks (higher vs. lower; Early: median_*diff*_ = −0.06, 95% HDI [−0.22, 0.10]; Late: median_*diff*_ = 0.00, 95% HDI [−0.12, 0.12]; Supplementary Fig. 5b, d).

## Discussion

People often change their evaluations and opinions upon learning about their peers’ evaluations and choices, i.e., social learning^7–9^. Moreover, sleep impacts social and non-social decision-making^46–49^. Combining the social learning paradigm with sleep-based targeted memory reactivation (TMR), we investigated whether reactivating the daytime social learning experience during non-rapid-eye-movement (NREM) sleep could further promote social learning-induced evaluation updating. Although TMR did not modulate social learning, social learning-induced evaluation updating became enlarged following overnight sleep for both cued and uncued snacks. Examining sleep EEG activity showed that the cue-elicited delta-theta (1–8 Hz) power and the overnight N2 spindle density predicted the overnight evaluation updating of cued but not uncued snacks. Together, we provided new evidence that neural activity indicating memory reactivation supports social learning and evaluation updating during sleep.

TMR benefits various types of learning by promoting sleep-mediated memory consolidation^22^. However, how TMR may benefit social learning remains largely unknown. A previous study endeavored to influence interpersonal trust via TMR, yet without sleep or TMR effect^50^. Here, although we did not find a significant TMR behavioral effect, cue-elicited delta-theta power and the overnight N2 spindle density predicted social learning-induced evaluation updating for cued snacks. Mounting evidence has suggested that delta-theta power characterizes memory reactivation during sleep^42,45,51–53^. Our findings are thus consistent with this research demonstrating the beneficial role of cue-elicited delta-theta power in evaluation updates^31,39^ and long-term memory maintenance^34–36^.

Sleep spindles support memory re-processing during sleep^34,41,51,54^. Here, although cue-elicited spindle activities did not predict evaluation updating, we found that the overnight N2 spindles were associated with overnight evaluation updating. This finding aligns with previous non-TMR sleep studies demonstrating that spontaneous N2 spindle density could predict memory consolidation^55–57^. However, overnight spindles only predicted cued but not uncued snacks, suggesting that TMR may bias overnight endogenous memory reactivation towards the cued snack^58^. Supporting this hypothesis, we found that the late-night (following TMR) N2 spindle density significantly predicted evaluation updating for cued snacks. While this explanation is tentative, future research could examine cue-elicited and spontaneous spindle activities to better understand the role of spindles in exogenous and endogenous memory reactivations^34,40,54^.

Our findings contribute to the theoretical understanding of how memory impacts evaluations in social learning and sleep^1,2^. We found that only when participants could correctly remember the feedback direction, they showed the social learning effect by following peers’ evaluations. Contrary to previous research that focused on memory interference that weakens memories^59^, our study aimed to change evaluation through sleep-mediated memory reactivation and consolidation. Together with previous TMR and sleep research on memory and evaluations^30,31^, we provided new evidence on how TMR and overnight sleep would also influence social learning-induced evaluation updating.

In addition to memory accuracies that capture episodic retrieval of peers’ evaluations, we also measured participants’ familiarity ratings towards the snacks. Intriguingly, we found that TMR increased familiarity with the cued snacks in the 3-day delayed session, which may influence the delayed evaluations. This finding aligned with well-established findings that people preferred familiar over unfamiliar snacks^60,61^ and the findings that merely re-playing snacks’ names during sleep could enhance people’s preference toward these snacks^31^. Notably, the TMR’s benefits in strengthening familiarity emerged in the delayed but not in the immediate test, which is consistent with recent evidence that TMR often showed delayed benefits^25,54^. One intriguing question that warrants future research is the respective impacts of episodic memory and familiarity on human evaluations and decision-making, and how sleep may influence different retrieval processes that support decision-making.

Limitations and future directions shall be discussed. First, while we found overnight evaluation updating for both cued and uncued snacks, item-level cue-elicited EEG activity only predicted evaluation updating for cued snacks. One possibility is that the reactivation of cued snacks may generalize to uncued snacks, given that they share the same learning context^35,62,63^. Future research shall test whether and when generalization occurred during TMR and sleep. Second, the classic social learning paradigm adopted here involved passive observation of peers’ evaluations in laboratory settings. Given that social learning often happens during real-life interpersonal interactions^64,65^, future research shall examine the role of sleep and TMR in consolidating more realistic social learning experiences. Lastly, while people are intrinsically motivated to follow peers’ opinions given the universal need to seek social belongingness^66,67^, our study did not manipulate motivations involved in many social learning scenarios^68^. Given that motivation could bias memory reactivation during sleep^69,70^, future research shall consider manipulating motivational processes during social learning, and examine how motivation interacts with sleep and memory reactivation to change behavior.

In conclusion, we found that the social learning-induced evaluation updating became more pronounced after sleep, irrespective of memory cueing during sleep. Sleep EEG activity, such as the cue-elicited delta-theta power and the overnight N2 spindle activity, supported the evaluation updating for the cued snacks. Our research contributes to the theoretical understanding of memory-based evaluation by highlighting the significance of offline sleep-mediated memory reactivation processes. Considering social learning can influence moral decision-making^12^ and healthy behavior^11,71–73^, using TMR and sleep in conjunction with social learning may offer insights into fostering adaptive behaviors in social and healthy contexts.

## Methods

### Participants

We recruited 45 participants from a local university (35 females; Age, Mean = 22.98, S.D. = 2.81). Participants were excluded from subsequent behavioral and EEG analysis if the auditory cues were played fewer than four rounds (*n* = 9) or due to technical problems during EEG recording (*n* = 2), resulting in 34 participants being included in the analyses. All participants were native Chinese speakers, right-handed, not color-blind, and had normal or correct-or-normal vision. In addition, they reported good sleep qualities without any history of neurological, psychiatric, or sleep disorders. All participants provided written informed consent prior to the participation and were debriefed and compensated after they completed the study. This research was approved by the Human Research Ethics Committee of the University of Hong Kong (HREC No. EA1904004).

### Stimuli

We selected 48 snack images from the snack and food images database^74,75^. Spoken names of snacks were generated in English using the Microsoft Azure Text-to-Speech function (language = “en-GB”). The 48 snacks were then allocated to one of six experimental conditions based on each participant’s baseline evaluation (i.e., the preference rating before the social learning). To do this, all 48 snacks were first sorted in descending order based on the baseline ratings and were subsequently divided into eight subgroups following this ranked order, each consisting of six snacks. For instance, snacks in this first subgroup would rank from first to sixth, while snacks in the second subgroup would rank from seventh to twelfth, and so on. Next, in each of the eight subgroups, the six snacks were randomly assigned to six experimental conditions from the 2 (TMR: cued vs. uncued) by 3 (social feedback from peers: lower vs. consistent vs. higher) design. This procedure resulted in eight items in each of the six experimental conditions, with baseline preferences and familiarity ratings not significantly different between different conditions (*p*s > 0.087; see Supplementary Table 1 for details).

### Design and procedure

All tasks were programmed and presented by PsychoPy (2020.1.3)^76^. Participants visited the lab twice, separated by three days (Fig. 1a).

During the first lab visit, participants arrived at the lab at around 20:00. After cleaning up and the EEG setup, participants completed the Interpersonal Reactivity Index (IRI)^77^, the Balanced Inventory of Desirable Responding (BIDR)^78^, and provided demographical information. Participants completed the following tasks in order. First, participants completed a psychomotor vigilance task (PVT, to measure alertness), a cue familiarization task (to get familiar with auditory cues and snack images), and an evaluation task (to indicate their baseline preferences for snacks). Second, participants performed a social learning task in which they learned about their peers’ evaluation of snacks (i.e., snack-peers’ rating associations) while hearing the spoken names of the snacks (i.e., memory reminders). Following the social learning task, participants completed the following post-learning tests: an affect misattribution procedure (AMP) task (to measure spontaneous evaluation), a speeded choice task (to measure choice), another evaluation task, and a cued recall task (to measure memories for peers’ ratings). Upon finishing these tasks, participants went to the overnight sleep session, wherein trained experimenters administered the TMR during NREM sleep.

After ~8 h of bedtime (12 a.m. to 8 a.m.), participants woke up and had breakfast. After ~20 min of refreshing up, participants’ vigilance levels were assessed again, followed by AMP, speeded choice task, evaluation task, and cued recall task. Three days later, participants returned to the same lab and completed the same set of tasks.

To test whether vigilance levels might differ across phases, participants completed a 5-minute Psychomotor Vigilance Task (PVT) at the beginning of each phase. During the PVT, a fixation was first presented on the center of the screen with a jitter duration of 2–10 s. Next, a counter starting from 0 would replace the fixation. Participants shall press the button as soon as they detect the changes. Their response times (RTs) were presented on the screen as the performance feedback. We found no significant RT differences across phases, *F* (1.62, 53.41) = 1.78, *p* = 0.183, ${\eta }_{G}^{2}$ = 0.01, suggesting no significant differences in vigilance levels across phases.

Following the PVT, participants were familiarized with the spoken names of the snacks in the cue familiarization task. Each trial started with a 0.3 s fixation, followed by a snack image (see Fig. 1 for examples), which was presented on the center of the screen for 2 s, accompanied by its spoken name (i.e., “Combos”) being played via an external speaker. The inter-trial interval (ITI) was 1 s. The task included three blocks, each containing all 48 snacks being randomly presented.

To assess participants’ evaluation of the snacks, we asked participants to rate their preference and familiarity with all 48 snacks four times: at pre-learning (baseline), post-learning, post-TMR, and 3-day delayed phases (Fig. 1b). In the evaluation task, each trial began with a 0.3 s fixation, followed by the presentation of a snack image on the screen. Using a blue triangle presented on the screen, participants then evaluated their preference for the item on a 1–11 scale (1 = Extremely Unwanted, 11 = Extremely Wanted) and their familiarity with the item (1 = Extremely Unfamiliar, 11 = Extremely Familiar). Next, we calculated the evaluation updating (ΔEvaluation) as outcome measures by subtracting the rating between every two phases, including post-learning minus pre-learning (immediate ΔEvaluation), post-TMR minus post-learning (overnight ΔEvaluation), delayed minus post-learning (delayed ΔEvaluation, Fig. 1a).

During the social learning task, participants learned their peers’ evaluations (Fig. 1c). Participants were informed that their peers were students from the same university. The learning included 240 trials in 5 blocks, with each block containing all 48 snacks. Each trial started with a blank screen (1.2–1.8 s), followed by a fixation cross (0.5 s). The snack image was then presented in the center of the screen for 1.5 s, together with participants’ baseline evaluation as indicated by a triangle on the preference rating scale. The scale disappeared on the screen, leaving the same snack image on the screen for 1.5 s as a buffer. Afterward, the peer’s rating was indicated by a circle on the same preference rating scale for 3 s, while the spoken name of the snack was aurally played (~1 s) to be linked with the peers’ preference ratings. Following a 1.5 s blank screen, with only snack images being presented on the screen, participants rated the preference again (3 s maximum) using the mouse. Note that the peer ratings feedback was pre-programmed for each participant: feedback was either consistent, higher, or lower than participants’ pre-learning baseline ratings. In the higher or lower conditions, the group ratings would be 1, 2, or 3 points above or below the participants’ initial ratings, respectively. To increase the authenticity of the feedback, the chance of 3-point difference feedback was half of the probability of receiving 1 or 2-point difference feedback. We divided 48 snacks into the six experimental conditions to ensure the baseline preference ratings were comparable across conditions (for details, see Stimuli).

To measure the implicit evaluation for snacks, we performed the Affect Misattribution Procedure (AMP)^79^ in the post-learning, post-TMR, and delayed tests. Each trial of the AMP task started with a 0.3 s fixation, followed by a snack image serving as a prime. The snack image was shortly presented for 75 ms, followed by a 925 ms blank screen. Afterward, a Tibetan character was presented on the screen for 0.1 s and replaced by a mosaic image as a mask. Participants decided as soon as possible whether the target character was pleasant (“A”) or unpleasant (“L”). The AMP task contained six blocks. Forty-eight snacks were randomly presented in each block. We then calculated the update of implicit evaluation (ΔImplicit evaluation) by subtracting the percentage of choosing “pleasant” between post-TMR/delayed and post-learning phases at the item level.

In the Speeded Choice Task, participants made speeded choices (purchase or not) toward the snacks using their own compensation in the speeded choice task. Participants completed this task three times: in the post-learning, post-TMR, and delayed tests. Each trial started with a 0.3 fixation, followed by a snack image presented on the screen for 1.5 s maximum. Participants were required to respond as soon as possible whether they would like to purchase the snack or not (“A” for yes, “L” for no). The speeded choice task contains three blocks, with 48 snacks randomly presented in each block. We then calculated the choice updating (Δ%Choose) by subtracting the percentage of choosing “Yes” between post-TMR/delayed and post-learning phases at the item level.

In the Cued Recall Task, we assessed participants’ memory of their peers’ ratings for each snack. Participants shall recall and indicate their peers’ ratings in the post-learning, post-TMR, and delayed phases. In the post-learning tests, the cued recall task contained two blocks: a test with a feedback block and a test without feedback block. In the feedback block, each trial began with a 0.3 s fixation, followed by a snack image and a preference rating scale being visually presented, accompanied by the spoken name of the snack. Participants clicked on the scale to indicate their peers’ preference rating. Following a 1 s blank screen, the correct ratings were presented as feedback, together with the same snack image accompanied by its spoken name aurally played. In the no-feedback block, trials were similar to those in the feedback block, except no feedback was presented. In both the post-TMR and delayed phases, participants indicated their memories of peers’ ratings for each snack without feedback.

Memory error was defined as the absolute difference between participants’ recall of the feedback and the presented feedback rating. We also coded participants’ memory accuracy as follows: If participants’ recollection of peers’ ratings aligned with the feedback directions (e.g., higher, lower, consistent), the memory was deemed correct. Conversely, the memory was deemed incorrect. Thus, accuracy was coded regardless of the numerical discrepancies between the peers’ ratings and the recall.

### TMR during NREM sleep

Half of the spoken names of the snacks (24 out of 48, e.g., “Combos”) and eight additional spoken names of food items (e.g., “Celery”) were played during the TMR. These eight stimuli were never presented before the TMR and were not paired with any peers’ ratings, thus serving as non-memory control cues. Throughout the night, pink noise was played as the background noise. Well-trained experimenters monitored the EEG brainwaves and identified the sleeping stages for TMR administration. For online sleep monitoring, F3/F4, C3/C4, P3/P4, O1/O2, EOG, and EMG, with online reference at CPz, were selected. Upon detection of stable slow-wave sleep for at least 5 minutes, the names of the snacks were played via a loudspeaker placed above the participant’s head. In each block of the TMR, all 32 cues (24 snack cues and eight control cues) were randomly played (~1 s) with an inter-stimulus interval (ISI) of 4 s. A 30-s interval separated each round of playing. The TMR phase was terminated when 20 cueing rounds were completed or reached 2 a.m., whichever came first. Cueing was immediately paused when participants showed signs of micro-arousal or awakening and entered N1 or REM sleep. Cueing would be resumed when participants returned to stable slow-wave sleep. Participants were excluded if they received fewer than 4 TMR rounds (*n* = 9). Accuracies of TMR cueing were validated by comparing the cueing time with offline sleep staging results using the YASA toolbox (0.6.1)^80^, which confirmed that the majority of cues were played during the N3 sleep stage (Mean ± S.D., 92.28 ± 18.40%).

### EEG acquisition

Continuous EEGs were recorded with an eego amplifier and a 64-channel gel-based waveguard cap based on an extended 10–20 layout (ANT Neuro, Enschede, and Netherlands). The online sampling rate was 500 Hz, with CPz as the online reference and AFz as the ground electrode. The horizontal electrooculogram (EOG) was recorded from an electrode placed 1.5 cm to the left external canthus. The impedance of all electrodes was maintained below 20 kΩ during the recording. During sleep, two additional electrodes were attached to both sides of the chins to measure electromyography (EMG) with a bipolar reference.

### EEG preprocessing

Sleep EEG was processed offline using custom Python (3.8.8) scripts and MNE-Python (0.23.4)^81^. To facilitate subsequent EEG preprocessing and analyses, the overnight EEG was cropped from 300 s ahead of the first and 300 s after the last TMR cue. Unused channels (EOG, M1, and M2) were removed from the cropped EEG data. Cropped raw EEG was notch-filtered at 50 Hz and next filtered with a bandpass filter of 0.5–40 Hz. Afterward, the EEG was downsampled to 250 Hz. Bad channels were then visually detected, removed, and interpolated. The EEG data were next re-referenced to the whole-brain average, followed by segmentation into [−15 s to 15 s] epochs relative to the onset of the cue for spindle probability analysis. Bad epochs were then visually detected and removed from further analyses. Artifacts-free EEG data were further segmented into [−2 s to 6 s] epochs for time-frequency analysis. The number of remaining epochs for each condition is provided in Supplementary Table 2. The overnight continuous EEG data were also retained for sleep staging and overnight spindle detection.

### Time-frequency analysis

In the time-frequency analysis, wavelets transformation with variance cycles (three cycles at 1 Hz in length, increasing linearly with frequency to 15 cycles at 30 Hz) was applied to the [−2 s to 6 s] epochs to compute time-frequency representation (TFR) for the EEG on each of the 61 channels. Subsequently, epochs were further segmented into [−1 s to 4 s] epochs to eliminate edge artifacts. The trial-level spectral power was normalized (Z-scored) using [−1 s to −0.2 s] baseline of the averaged spectral power of all trials. We then performed statistical tests (see Statistical Analysis for details) on the averaged power within the nine pre-defined fronto-central channels (F1/2, Fz, FC1/2, FCz, C1/2, Cz). These nine channels were selected per previous studies examining auditory/memory processing during sleep^82,83^.

### Offline automated sleep staging

The offline sleep staging was conducted with the YASA toolbox (0.6.1)^80^ implemented in Python (3.8.8). Raw overnight continuous EEG data were re-referenced to FPz according to the YASA recommendation. Sleep staging was based on C4 (or C3 if C4 was marked as a bad channel) and EOG (see Supplementary Table 3 for sleep stage information).

### Spindle detection

The automated spindle detection was implemented in the YASA toolbox (0.6.1)^80^. We applied three thresholds in identifying a spindle: 1) relative power, the 11-16 Hz sigma power) relative to the total power in the 1-30 Hz broadband frequency, 2) correlation, the correlation between sigma-filtered signal and broadband signal, and 3) moving root mean square (RMS) of the sigma-filtered signal. At Cz (or C3 if Cz was marked as a bad channel). We detected overnight N2 spindles (relative power = 0.2, correlation = 0.65, RMS = 1.5)^84,85^ and N3 spindles (relative power = None, correlation = None, RMS = 1.5)^82,86^. The results related to N3 spindles are provided in Supplementary Fig. 6. For cue-elicited spindles, given that 92.28% of our cues were played during the N3, we used the same N3 spindle parameters to artifact-free [−15 s to 15 s] epochs relative to the cue onset. We adopted different spindle detection parameters for the N2 and N3 separately because N2 and N3 showed distinct EEG characteristics. Specifically, N3 sleep is characterized by high amplitude 0.5–4 Hz delta-wave activity, while N2 sleep is characterized by a burst of spindle activity and the K-complex, among ongoing theta activity^87,88^. Upon detection of individual spindles, we calculated the N2 spindle density using the following formula^55–57^:

1 $${\rm{Spindle}}\;{\rm{density}}\,\left({{N}}2\right)=\frac{{\rm{The}}\;{\rm{number}}\;{\rm{of}}\;{\rm{spindles}}\; {\rm{detected}}\left({{N}}2\right)}{{\rm{Length}}({{N}}2/\min )}$$

For cue-elicited spindles within the [−15 s to 15 s] epochs relative to cue onset, the algorithm generated a series of 0/1 binary values to indicate spindle presence or absence for each 4 ms timepoint. The cue-elicited spindle probability was next determined by computing the proportion of detected spindles across trials at each timepoint^34,82,89^. Finally, the epochs were further segmented into [−1 to 5 s] epochs.

### Statistical analysis

First, we investigated the impact of social learning and TMR on changes in evaluation, implicit evaluation, speeded choice, and memory error. We conducted repeated-measure ANOVA with R (4.2.2) and the afex package (1.2.1) implemented in R. We further examined the effects of social learning, TMR, and subsequent memory on evaluation updating. Due to the limited number of trials after separating trials into correctly vs. incorrectly remembered, we adopted an item-level linear mixed model. To deal with the singular fitting problem, we chose a Bayesian linear mixed model (BLMM) with R using the brms package (2.20.4)^90^. Since evaluations were only tested once in each phase, the evaluation updating at the item level is discrete (from -8 to 8). Therefore, we adopted a cumulative distribution in the BLMM and transformed the ΔEvaluation into ordinal-level data. The following BLMM was applied:

2 $$\begin{array}{l}\Delta {\rm{Evaluation}} \sim {\rm{TMR}}* {\rm{Feedback}}* {\rm{Subsequent}}\;{\rm{Memory}}\\+(1+{\rm{Feedback}}* {\rm{Subsequent}}\;{\rm{Memory}}\;{\rm{SubjectID}})\end{array}$$

Next, we investigated whether cues would elicit significantly different EEG power changes and spindle probability. We employed a cluster-based two-tailed one-sample permutation test, implemented in the MNE toolbox with 1000 randomizations and a statistical threshold of 0.05.

To quantify the relationship between cue-elicited power and overnight and delayed ΔEvaluation, we continued to utilize item-level BLMM. The cue-elicited power was extracted from the significant clusters at the item level. We also adopted a cumulative distribution and transformed the ΔEvaluation to ordinal-level data. Because we considered that the cueing repetition could impact the signal-to-noise ratio of EEG data, we took the repetition number (N) as a covariate. The following BLMM was employed:

3 $$\Delta {\rm{Evaluation}} \sim {\rm{Power}}* {\rm{Feedback}}+{\rm{N}}+(1+{\rm{Power}}* {\rm{Feedback\; SubjectID}})$$

The same item-level BLMM was employed to investigate the relationship between cue-elicited spindle probability and evaluation updating:

4 $$\begin{array}{l}\Delta {\rm{Evaluation}} \sim {\rm{Spindle}}\;{\rm{Prob}}.* {\rm{Feedback}}+{\rm{N}}\\+(1+{\rm{Spindle}}\;{\rm{Prob}}.* {\rm{Feedback}}|{\rm{SubjectID}})\end{array}$$

We were also interested in the impact of overnight spindle density on the overnight and delayed ΔEvaluation. For this purpose, we conducted the following subject-level BLMMs on the overnight and delayed ΔEvaluation respectively:

5 $$\begin{array}{l}\Delta {\rm{Evaluation}} \sim {\rm{Spindle}}\;{\rm{Density}}* {\rm{Feedback}}* {\rm{TMR}}\\+(1+{\rm{Spindle}}\;{\rm{Density}}* {\rm{TMR}}|{\rm{SubjectID}})\end{array}$$

Statistical inferences for the BLMM were based on the 95% highest density interval (HDI) of the posterior distribution. Effects were considered significant if the 95% HDI did not encompass 0. Note that we focused on evaluation updating in the higher and lower conditions, wherein participants were expected to change their evaluations. It is important to note that employing Bayesian statistics mitigates concerns of multiple comparisons^91,92^, which allows a more straightforward interpretation of results across different comparisons in our study (e.g., cued vs. uncued).

### Reporting summary

Further information on research design is available in the Nature Research Reporting Summary linked to this article.

### Supplementary information

Supplementary Information

Reporting Summary

## Data Availability

Preprocessed data are available on the Open Science Framework (OSF) at https://osf.io/t96z5. Supplementary materials are available online.

## Code availability

Analysis scripts are available on the Open Science Framework (OSF) at https://osf.io/t96z5.
