# Supplementary material for: Modulating social learning-induced evaluation updating during human sleep
Source: NPJ Sci Learn. 2024 Jul 7;9:43. doi: 10.1038/s41539-024-00255-5 (PMC11227583; doi:10.1038/s41539-024-00255-5)
Supplement: Supplementary file 1 — Supplementary Information [file 41539_2024_255_MOESM1_ESM.pdf]

**Supplementary Information for**

# Modulating social learning-induced evaluation updating during human sleep

Danni Chen[1], Tao Xia[1], Ziqing Yao[1], Lingqi Zhang[1] Xiaoqing Hu[1,2*]

1, Department of Psychology, The State Key Laboratory of Brain and Cognitive Sciences, The University of Hong Kong, Hong Kong SAR, China

2, HKU-Shenzhen Institute of Research and Innovation, Shenzhen, China

***Corresponding author:**

X.H., xiaoqinghu@hku.hk

**This file includes:**

Supplementary Figures 1 to 6

Supplementary Tables 1 to 3

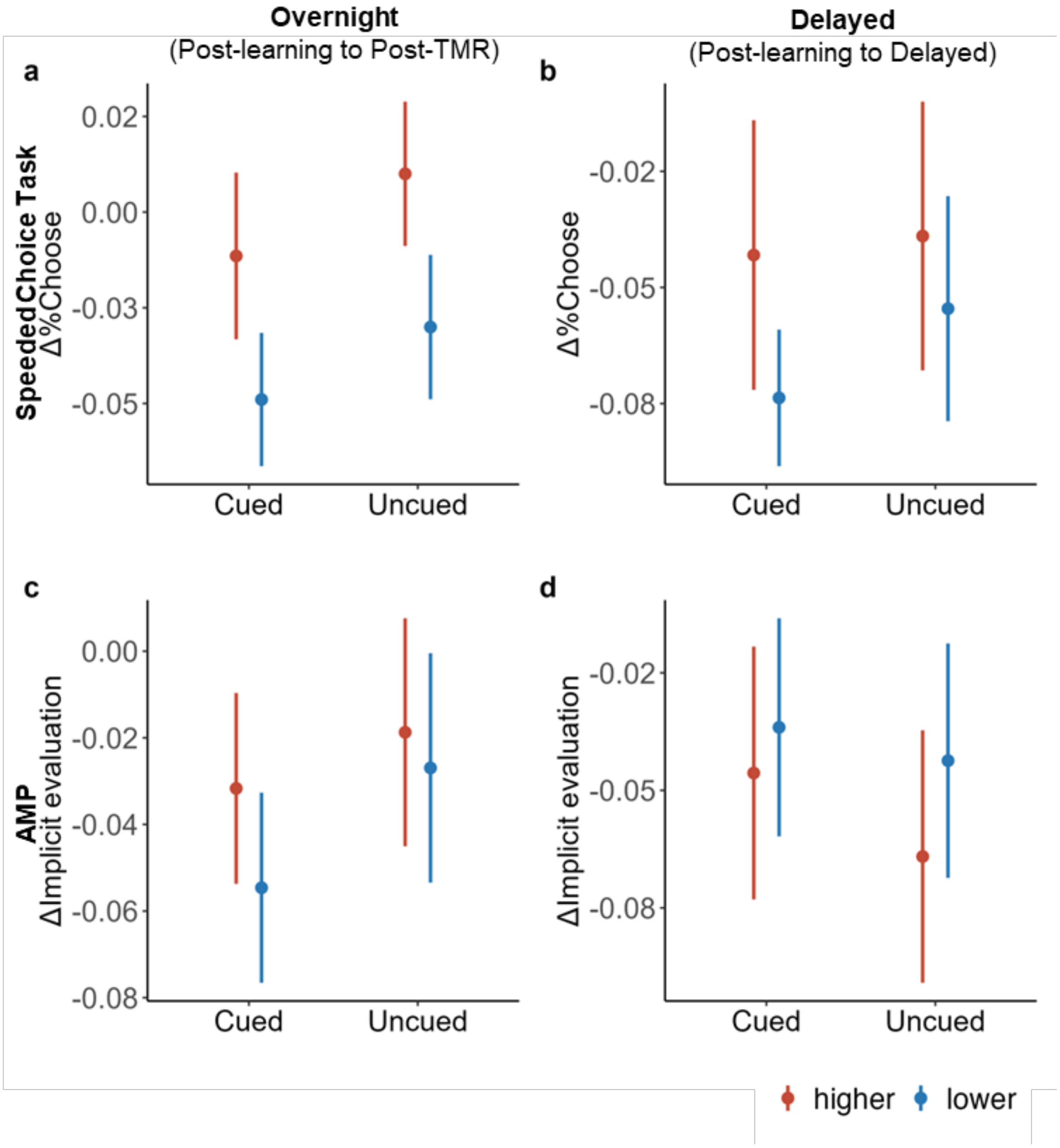


**Supplementary Figure 1 | Behavioral Results in the Speeded Choice and AMP task.** Impact of TMR and social learning on Δ%Choose in the speeded choice (**a**) from post-learning to post-TMR phase, and (**b**) from post-learning to delayed phase. Impact of TMR and social learning on ΔImplicit evaluation in the AMP task (**c**) from post-learning to post-TMR phase, and (**d**) from post-learning to delayed phase. The error bars indicate the standard error of the mean (S.E.M.)

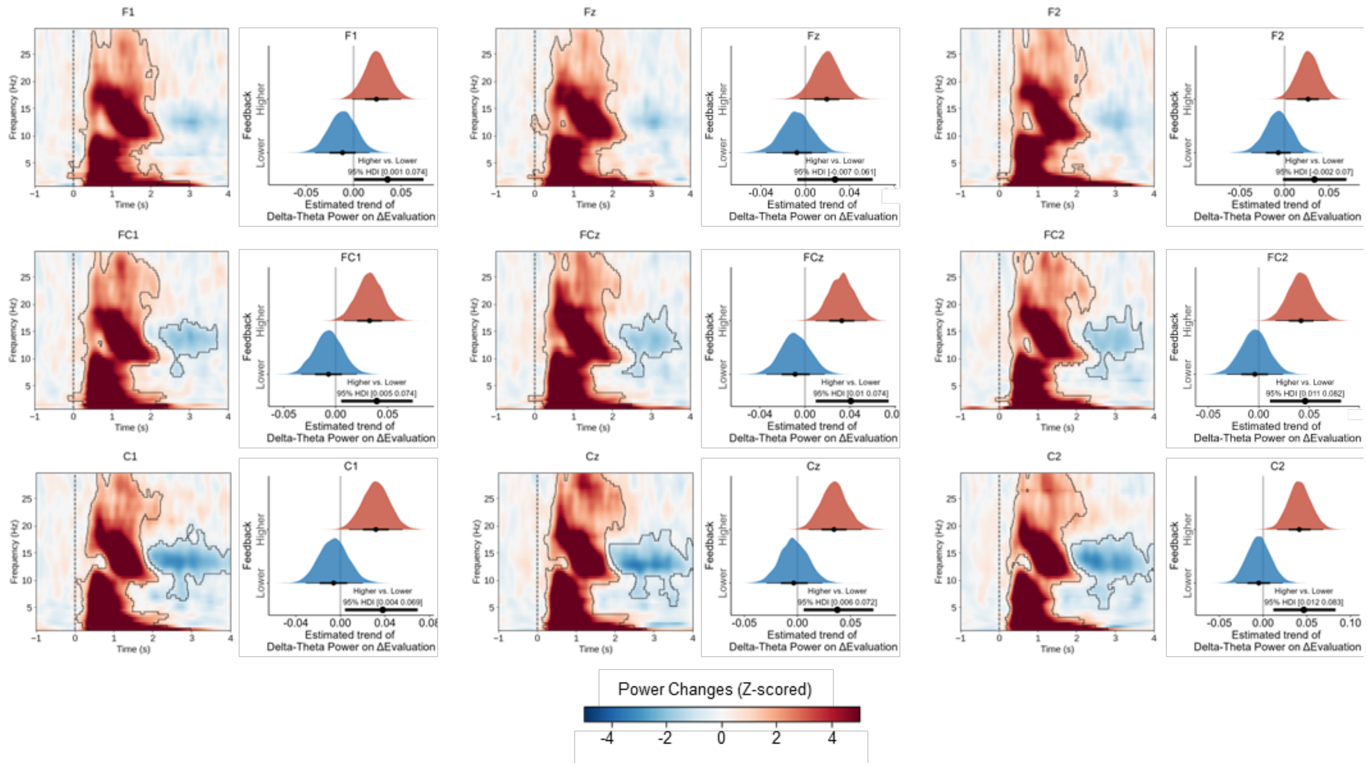


**Supplementary Figure 2 | Cue-elicited EEG Power of Individual Electrode and ΔEvaluation.**

We conducted two-tailed one-sample permutation tests on the EEG power of each of the 9 pre-selected fronto-central channels (F1/2, Fz, FC1/2, FCz, C1/2, Cz). The findings are largely in line with the results of the average power calculated across these fronto-central channels. Significant clusters ($p_{\text{cluster}} < .05$) are highlighted by the contour. Next, for consistency, we extracted the item-level delta-theta power (1-8 Hz) on each of the nine channels within the 0 to 2-second post-cue time window of the earlier cluster. The earlier cluster was identified by the cluster-based one-sample permutation test on the average power across the nine fronto-central channels. We employed item-level Bayesian Linear Mixed Models (BLMMs) using the same model as the main text. In this model, we included delta-theta power and feedback (higher vs. lower) as fixed factors and the repeated times of each item as a covariate. The dependent variable was overnight ΔEvaluation. Our analyses consistently revealed a significantly higher slope between delta-theta power and ΔEvaluation for the higher feedback condition compared to the lower feedback condition in most fronto-central channels (C1/2, Cz, FC1/2, FCz, F1). The black horizontal line below the red (higher) and blue (lower) density plots indicated the 95% highest density interval (HDI) for the higher and lower feedback conditions, respectively. The bottom black horizontal line indicates the difference in higher vs. lower feedback conditions. The dot indicates the median. If the 95% HDI did not encompass 0, the result would be considered significant.

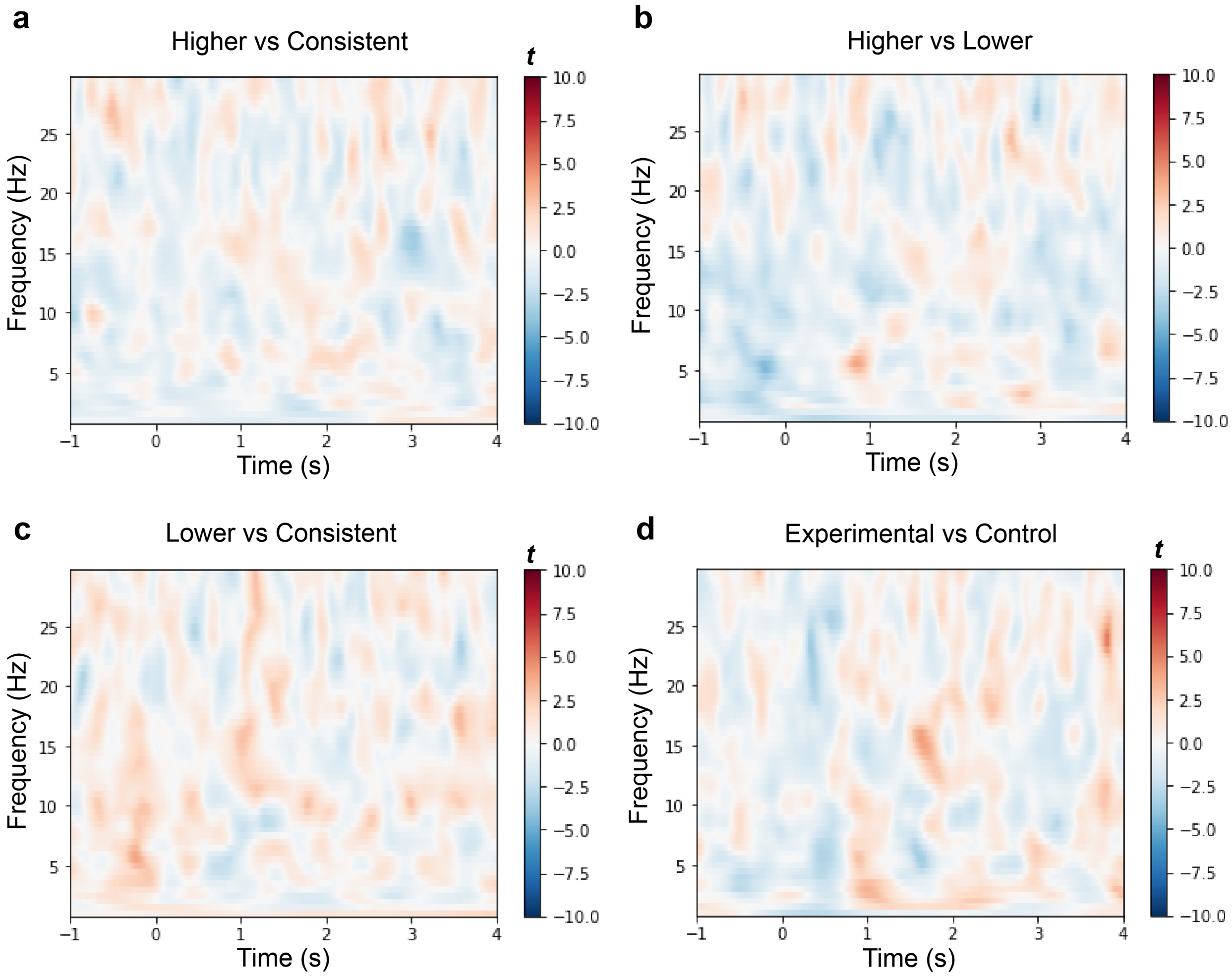


**Supplementary Figure 3 | Cue-elicited EEG Power.**
Cue-elicited EEG power comparing (**a**) "Higher" cue vs. "Consistent" cue, (**b**) "Higher" cue vs. "Lower" cue, and (**c**) "Lower" cue vs. "Consistent" cue, and (**d**) experimental cue vs. control cues,. No significant cluster was found across all four comparisons ($p_{cluster}$s > .085).

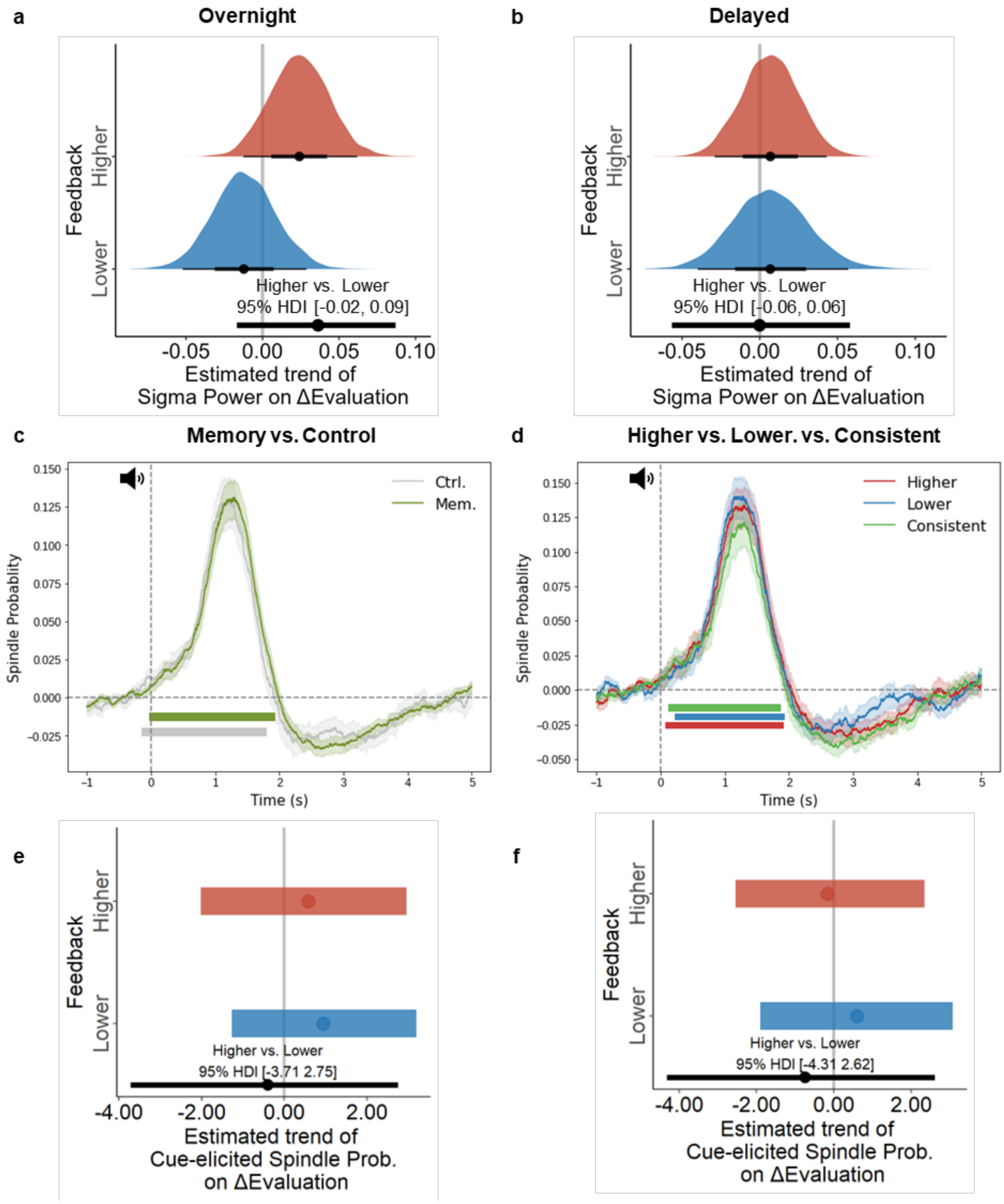


**Supplementary Figure 4 | Cue-elicited Spindle Activities and ΔEvaluation.**
(**a, b**) We examined whether cue-elicited sigma power (12-16 Hz) predicted overnight and delayed ΔEvaluation with item-level BLMMs. No significant effects were observed (Overnight: $median_{diff}$ = 0.04, 95% HDI [-0.02, 0.09]; delayed: $median_{diff}$ = -0.00, 95% HDI [-0.06, 0.06]). (**c, d**) Spindle Probability between Control and Memory cues (**c**) and among Higher, Lower, and Consistent cues (**d**). The shaded part indicated standard errors. The colored line indicated the significant clusters when comparing the spindle probability with the baseline probability

(Memory: $p_{cluster}$ = .001, -24~1920ms; Control: $p_{cluster}$ = .001, -148~1788ms; Higher: $p_{cluster}$ = .001, 76~1900ms; Lower: $p_{cluster}$ = .001, 228~1920ms; Consistent: $p_{cluster}$ =.001, 128~1868ms). We examined whether cue-elicited spindle probabilities predicted the changes in the (**e**) immediate and (**f**) delayed tests with item-level BLMMs. No significant effects were observed.

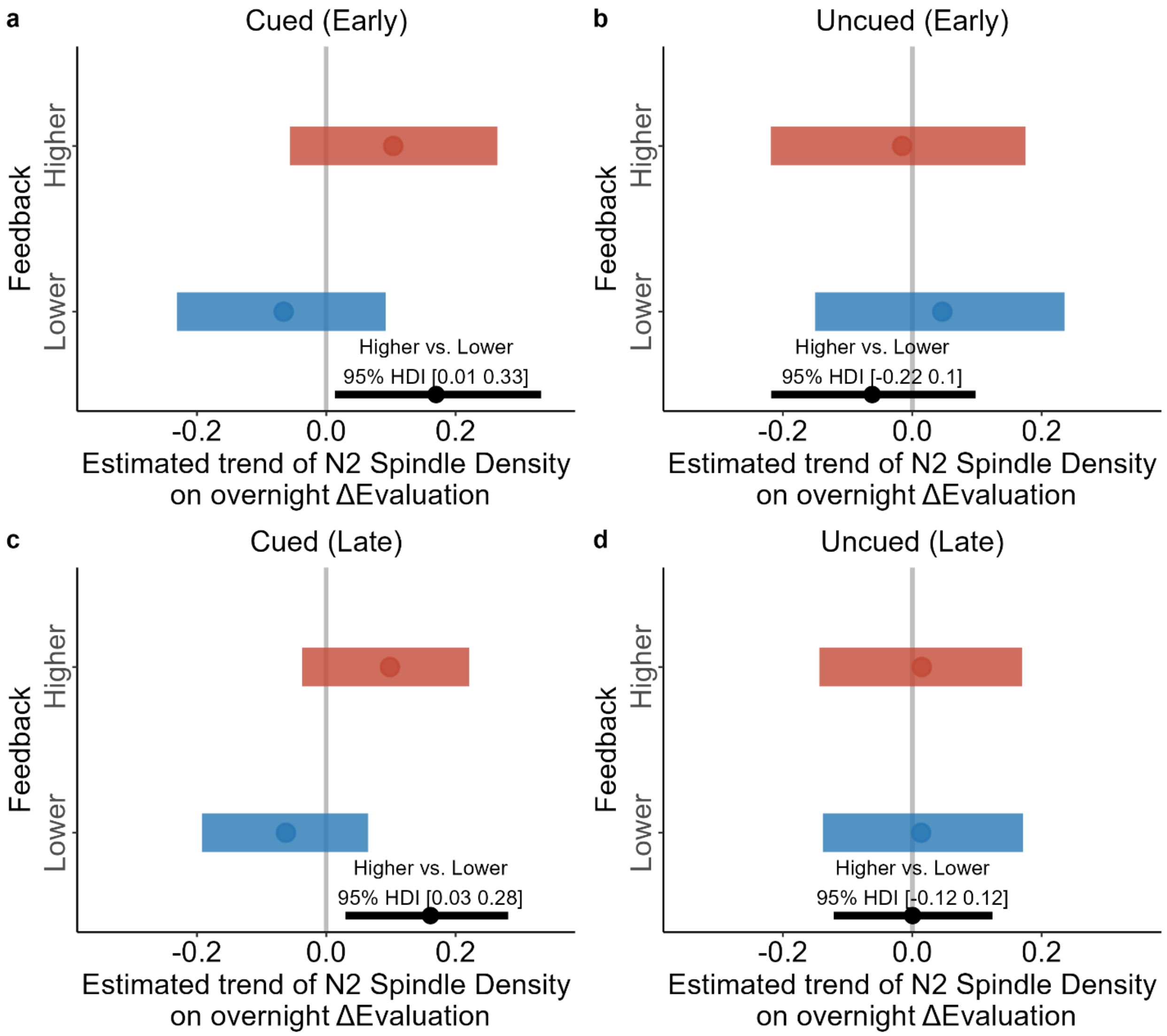


**Supplementary Figure 5 | Overnight N2 spindle density and cued snacks' overnight ΔEvaluation from post-learning to post-TMR phases.**
To understand the function of cueing-related and spontaneous spindle activities in the evaluation changes, we further explored whether spindle density during the early night (first three hours of sleep, overlapping with the TMR) and late night (after three hours until wakefulness, not overlapping with the preceding TMR) predicted overnight ΔEvaluation. In this model, we included stage (early vs. late), TMR (cued vs. uncued), feedback (higher vs. lower), and N2 spindle density as fixed factors. The results showed that both the (**a**) early and (**c**) late spindle density predicted the overnight ΔEvaluation of the cued snacks (higher vs. lower; Early: $median_{diff}$ = 0.17, 95% HDI [0.01, 0.33]; Late: $median_{diff}$ = 0.16, 95% HDI [0.03, 0.28]). In contrast, no significant effect of both the (**b**) early and (**d**) late spindle density in the overnight ΔEvaluation of the uncued snacks (higher vs. lower; Early: $median_{diff}$ = -0.06, 95% HDI [-0.22, 0.10]; Late: $median_{diff}$ = 0.00, 95% HDI [-0.12, 0.12]). The vertical gray lines correspond to 0. The horizontal red and blue lines indicated the 95% highest density interval (HDI) for higher and

lower feedback conditions, respectively. The bottom black horizontal line indicates the difference in higher vs. lower feedback conditions. The dot indicates the median. If the 95% HDI did not encompass 0, the result would be considered significant.

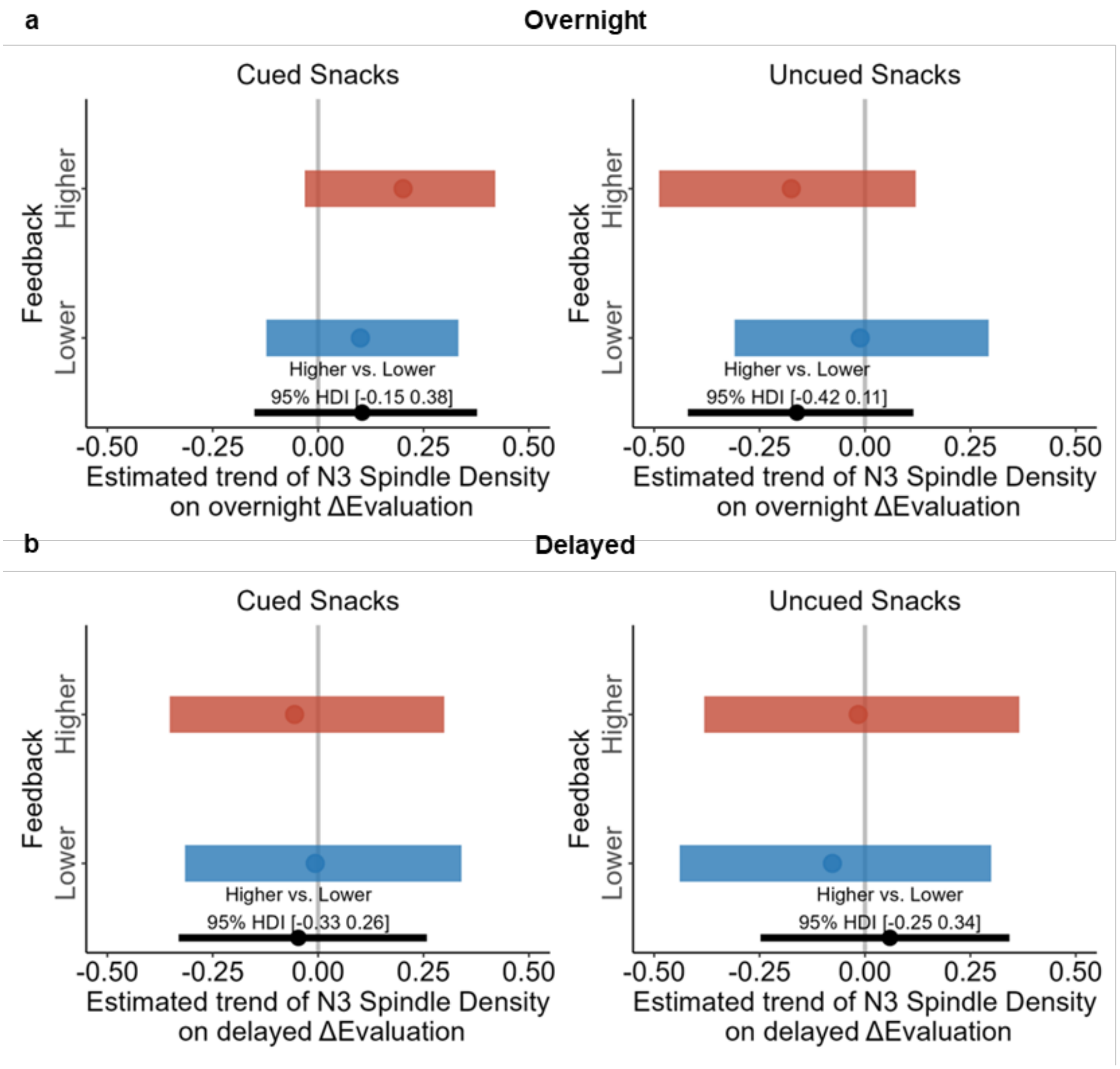


**Supplementary Figure 6 | The relationship between overnight N3 Spindle Density and ΔEvaluation.**

We explored whether overnight N3 Spindle Density predicted overnight and delayed ΔEvaluation by employing subject-level BLMMs. In this model, we employed TMR (cued vs. uncued), feedback (higher vs. lower), and N3 spindle density as fixed factors. (**a**) The N3 spindle density did not significantly predict overnight ΔEvaluation per directions of social feedback (higher vs. lower, cued snacks: $median_{diff}$ = 0.10, 95% HDI [-0.15, 0.38]; uncued snacks: $median_{diff}$ = -0.16, 95% HDI [-0.42, 0.11]). (**b**) The N3 spindle density did not significantly predict delayed ΔEvaluation for higher and lower feedback conditions differently (higher vs. lower, cued snacks: $median_{diff}$ = -0.05, 95% HDI [-0.33, 0.26]; uncued snacks: $median_{diff}$ = 0.06, 95% HDI [-0.25, 0.34]). The vertical gray lines correspond to 0. The horizontal red and blue lines indicated the 95% highest density interval (HDI) for higher and lower feedback conditions, respectively. The bottom black line indicates the difference in higher vs. lower feedback

conditions. The circle indicates the median point. If the 95% HDI did not encompass 0, the result would be considered significant.

**Supplementary Table 1**

*Baseline Preference and Familiarity (Mean [S.D.]) Across Conditions*

| Measurements | Preference | Familiarity |
|---|---|---|
| *Cued* | | |
| Higher | 6.05 (1.02) | 5.56 (1.25) |
| Consistent | 6.07 (1.03) | 5.59 (1.25) |
| Lower | 6.09 (0.98) | 5.90 (1.32) |
| *Uncued* | | |
| Higher | 6.05 (1.02) | 5.67 (1.08) |
| Consistent | 6.13 (1.06) | 5.74 (1.17) |
| Lower | 6.09 (1.04) | 5.84 (1.08) |
| *Statistics* | | |
| Main effect of TMR | $F$ (1, 33) = 0.86, $p$ = .361 | $F$ (1, 33) = 0.22, $p$ = .644 |
| Main effect of feedback | $F$ (1.90, 62.72) = 2.58, $p$ = .087 | $F$ (1.83, 60.24) = 1.89, $p$ = .163 |
| Interaction effect | $F$ (1.86, 61.52) = 0.87, $p$ = .419 | $F$ (1.78, 58.90) = 0.30, $p$ = .715 |

**Supplementary Table 2**

*Remained Epoch Numbers in TMR*

| Condition | Mean | S.D. |
|---|---|---|
| Higher | 113.82 | 31.21 |
| Lower | 113.91 | 31.26 |
| Consistent | 113.97 | 30.84 |
| Control | 114.18 | 30.94 |
| In total | 455.88 | 124.25 |

**Supplementary Table 3**

*Sleep Measurements*

| Measurement | Mean | S.D. |
|---|---|---|
| TIB | 483.59 | 26.27 |
| SPT | 467.96 | 33.03 |
| WASO | 25.99 | 24.32 |
| TST | 441.97 | 41.87 |
| N1 | 20.72 | 9.60 |
| N2 | 215.22 | 26.88 |
| N3 | 102.68 | 26.34 |
| REM | 103.35 | 24.26 |
| NREM | 338.62 | 32.17 |
| SOL | 14.10 | 17.64 |
| Lat_N1 | 23.35 | 54.16 |
| Lat_N2 | 19.28 | 18.33 |
| Lat_N3 | 28.15 | 20.25 |
| Lat_REM | 95.12 | 29.07 |
| %N1 | 4.77 | 2.33 |
| %N2 | 48.89 | 5.99 |
| %N3 | 23.10 | 5.11 |
| %REM | 23.24 | 4.64 |
| %NREM | 76.76 | 4.64 |
| conf_N1 | 0.58 | 0.05 |
| conf_N2 | 0.84 | 0.04 |
| conf_N3 | 0.89 | 0.04 |
| conf_R | 0.83 | 0.06 |
| SE | 91.37 | 6.73 |
| SME | 94.41 | 5.20 |
| Stability | 0.92 | 0.02 |

*Note*: TIB = Time in Bed. SPT = Sleep Period Time. WASO = Wake After Sleep Onset. TST = Total Sleep Time. N1, N2, N3, and REM: Sleep stages duration. NREM = N1 + N2 + N3. SOL = Sleep Onset Latency. Lat_N1, N2, N3, REM: latencies of sleep stages from the beginning of the record. %(N1, N2, N3, REM): Sleep stages duration expressed in percentages of TST. Conf_N1, N2, N3, REM: Confidence of sleep staging (Range = 0 to 1). S.E. = Sleep Efficiency. SME: Sleep Maintenance Efficiency. Stability = diagonal value in the transition matrix.
