# Supplementary material for: Modulating social learning-induced evaluation updating during human sleep
Source: NPJ Sci Learn. 2024 Jul 7;9:43. doi: 10.1038/s41539-024-00255-5 (PMC11227583; doi:10.1038/s41539-024-00255-5)
Supplement: Supplementary file 2 — Reporting Summary [file 41539_2024_255_MOESM2_ESM.pdf]

nature portfolio

Corresponding author(s): Xiaoqing Hu

Last updated by author(s): Jun 8, 2024

Reporting Summary

Nature Portfolio wishes to improve the reproducibility of the work that we publish. This form provides structure for consistency and transparency in reporting. For further information on Nature Portfolio policies, see our Editorial Policies and the Editorial Policy Checklist.

Statistics

For all statistical analyses, confirm that the following items are present in the figure legend, table legend, main text, or Methods section.

n/a	Confirmed	
☐	☒	The exact sample size (*n*) for each experimental group/condition, given as a discrete number and unit of measurement
☐	☒	A statement on whether measurements were taken from distinct samples or whether the same sample was measured repeatedly
☐	☒	The statistical test(s) used AND whether they are one- or two-sided<br>*Only common tests should be described solely by name; describe more complex techniques in the Methods section.*
☐	☒	A description of all covariates tested
☐	☒	A description of any assumptions or corrections, such as tests of normality and adjustment for multiple comparisons
☐	☒	A full description of the statistical parameters including central tendency (e.g. means) or other basic estimates (e.g. regression coefficient) AND variation (e.g. standard deviation) or associated estimates of uncertainty (e.g. confidence intervals)
☐	☒	For null hypothesis testing, the test statistic (e.g. *F*, *t*, *r*) with confidence intervals, effect sizes, degrees of freedom and *P* value noted<br>*Give P values as exact values whenever suitable.*
☐	☒	For Bayesian analysis, information on the choice of priors and Markov chain Monte Carlo settings
☐	☒	For hierarchical and complex designs, identification of the appropriate level for tests and full reporting of outcomes
☐	☒	Estimates of effect sizes (e.g. Cohen's *d*, Pearson's *r*), indicating how they were calculated

Our web collection on statistics for biologists contains articles on many of the points above.

Software and code

Policy information about availability of computer code

Data collection	PsychoPy (2020.1.3) for collecting behavioral data
Data analysis	Python (3.8.8), MNE-Python (0.23.4), R (4.2.2), afex (1.2.1), brms (2.20.4)

For manuscripts utilizing custom algorithms or software that are central to the research but not yet described in published literature, software must be made available to editors and reviewers. We strongly encourage code deposition in a community repository (e.g. GitHub). See the Nature Portfolio guidelines for submitting code & software for further information.

Data

Policy information about availability of data

All manuscripts must include a data availability statement. This statement should provide the following information, where applicable:

- Accession codes, unique identifiers, or web links for publicly available datasets
- A description of any restrictions on data availability
- For clinical datasets or third party data, please ensure that the statement adheres to our policy

Preprocessed data is available on the Open Science Framework (OSF) at https://osf.io/t96z5/?view_only=8bc5488d7d854455b19fe792e693410d.

Research involving human participants, their data, or biological material

Policy information about studies with human participants or human data. See also policy information about sex, gender (identity/presentation), and sexual orientation and race, ethnicity and racism.

Reporting on sex and gender	We recruited 45 participants from a local university (35 females; Age, Mean = 22.98, S.D. = 2.81).
Reporting on race, ethnicity, or other socially relevant groupings	All participants were native Chinese speakers.
Population characteristics	See above
Recruitment	Participants were recruited from a local university.
Ethics oversight	This research was approved by the Human Research Ethics Committee of the University of Hong Kong (HREC No. EA1904004).

Note that full information on the approval of the study protocol must also be provided in the manuscript.

Field-specific reporting

Please select the one below that is the best fit for your research. If you are not sure, read the appropriate sections before making your selection.

☐ Life sciences ☒ Behavioural & social sciences ☐ Ecological, evolutionary & environmental sciences

For a reference copy of the document with all sections, see nature.com/documents/nr-reporting-summary-flat.pdf

Behavioural & social sciences study design

All studies must disclose on these points even when the disclosure is negative.

Study description	We investigated how targeted memory reactivation (TMR) during sleep can influence the evaluations acquired by prior social learning. The empirical study included a two (TMR: cued vs. uncued) by three (feedback: higher, lower and consistent). We collected both behavioral responses and neural activities during the overnight sleep.
Research sample	45 Students from the University of Hong Kong (35 females; Age, Mean = 22.98, S.D. = 2.81). This sample is representative for participants without sleep problems.
Sampling strategy	The valid sample (34) is comparable to previous TMR studies.
Data collection	Participants' behavioral responses were collected by a computer, and their brainwaves during sleep were collected by EEGO amplifier and a 64-channel gel-based waveguard cap based on an extended 10-20 layout (ANT Neuro, Enschede, and Netherlands). No one was present besides the participants when they were doing the tasks. Experimenters were not blind to the study hypothesis during data collection.
Timing	September 1 2021 to October 19 2021
Data exclusions	Participants were excluded from subsequent behavioral and EEG analysis if the auditory cues were played fewer than four rounds (n = 9) or due to technical problems during EEG recording (n = 2), resulting in 34 participants being included in the analyses.
Non-participation	No participants dropped out.
Randomization	All participants went through all experimental conditions. All stimuli were randomly assigned to one of the six experimental conditions.

Reporting for specific materials, systems and methods

We require information from authors about some types of materials, experimental systems and methods used in many studies. Here, indicate whether each material, system or method listed is relevant to your study. If you are not sure if a list item applies to your research, read the appropriate section before selecting a response.

Materials & experimental systems

n/a	Involved in the study
☒	☐ Antibodies
☒	☐ Eukaryotic cell lines
☒	☐ Palaeontology and archaeology
☒	☐ Animals and other organisms
☒	☐ Clinical data
☒	☐ Dual use research of concern
☒	☐ Plants

Methods

n/a	Involved in the study
☒	☐ ChIP-seq
☒	☐ Flow cytometry
☒	☐ MRI-based neuroimaging

Plants

Seed stocks

Report on the source of all seed stocks or other plant material used. If applicable, state the seed stock centre and catalogue number. If plant specimens were collected from the field, describe the collection location, date and sampling procedures.

Novel plant genotypes

Describe the methods by which all novel plant genotypes were produced. This includes those generated by transgenic approaches, gene editing, chemical/radiation-based mutagenesis and hybridization. For transgenic lines, describe the transformation method, the number of independent lines analyzed and the generation upon which experiments were performed. For gene-edited lines, describe the editor used, the endogenous sequence targeted for editing, the targeting guide RNA sequence (if applicable) and how the editor was applied.

Authentication

Describe any authentication procedures for each seed stock used or novel genotype generated. Describe any experiments used to assess the effect of a mutation and, where applicable, how potential secondary effects (e.g. second site T-DNA insertions, mosiacism, off-target gene editing) were examined.
